# Risk Factors and Emerging Therapies in Amyotrophic Lateral Sclerosis

**DOI:** 10.3390/ijms20112616

**Published:** 2019-05-28

**Authors:** Natalia Nowicka, Jakub Juranek, Judyta K. Juranek, Joanna Wojtkiewicz

**Affiliations:** 1 Department of Pathophysiology, School of Medicine, Collegium Medicum, University of Warmia and Mazury, 10-900 Olsztyn, Poland; judytajuranek@gmail.com; 2 Institute of Psychology, Polish Academy of Sciences, Jaracza 1, 00-378 Warsaw, Poland; jakub.juranek@sd.psych.pan.pl

**Keywords:** ALS, risk factors, genetic factors, environmental factors, ALS treatment

## Abstract

Amyotrophic lateral sclerosis (ALS) is a fatal progressive neurodegenerative disease characterized by a permanent degeneration of both upper and lower motor neurons. Many different genes and pathophysiological processes contribute to this disease, however its exact cause remains unclear. Therefore, it is necessary to understand this heterogeneity to find effective treatments. In this review, we focus on selected environmental and genetic risk factors predisposing to ALS and highlight emerging treatments in ALS therapy. Of numerous defective genes associated with ALS, we focus on four principal genes that have been identified as definite causes of ALS: the *SOD1* gene, *C9orf72*, *TDP-43*, as well as the recently identified *TBK1*. We also provide up-to-date information on selected environmental factors that have historically been considered as key players in ALS development and pathogenesis. In parallel to our survey of known risk factors, we also discuss emerging ALS stem cell therapies and experimental medicines currently undergoing phase II and III clinical trials.

## 1. Introduction

Amyotrophic lateral sclerosis (ALS), also known as a motor neuron disease (MND), is a progressive neurodegenerative disorder that causes the degeneration of upper and lower motor neurons. It is the most common progressive and fatal neurodegenerative disease with an adult-onset, characterized by motor neuron degeneration in the primary motor cortex, brainstem, and spinal cord and progressive atrophy and weakness of the skeletal muscles [1]. There are two well-known forms of ALS: sporadic ALS (sALS), occurring in individuals with no family history of the disease, and familial ALS (fALS), occurring in at least two people in the same family. sALS comprises 85–90% of all cases. The remaining 10–15% of all cases account for the familial form of ALS [2,3]. The incidence of ALS differs slightly between genders; in the case of sALS, men are at a slightly higher risk of the disease ((3.0 per 100,000 person-years) as compared to women (2.4 per 100,000 person-years); however, in the case of fALS, the risk remains at similar levels for both genders. The age range at onset is 58–63 years for sporadic ALS and 47–52 years for familial ALS [4]. Nowadays, the division of ALS into two categories—familial or sporadic—is being challenged, as ALS has shown to be distinguished by a high differentiation of characteristics, i.e., phenotype, etiology, and progression. Multiple studies have shown that many environmental and genetic risk factors contribute to the onset of sporadic ALS; however, no single environmental or genetic factor has been clearly linked to sALS onset [5]. Hanspal and colleagues noticed a problem between the lack of evident difference in clinical symptoms between fALS and sALS patients, suggesting that a common molecular mechanism might be the basis of the disease [6]. Half of ALS patients die within 2–5 years of symptoms onset, generally from respiratory insufficiency [7,8]; however, some patients survive more than a decade from the initial diagnosis. 

Most patients (about 70%) present with limb-onset ALS, about 25% patients have bulbar-onset disease, and the rest of the patients (5%) manifest respiratory involvement or initial trunk [9] onset. 

Symptoms depend on which neurological region or motor level is affected. Bulbar-onset is associated with upper and/or lower motor neuron. Patients present with dysphagia (difficulty swallowing) and dysarthria (slurring of speech). Bulbar involvement depends on the motor neurons affected. Bulbar palsy (lower motor neuron) is associated with a deficiency in palatal movement with weakness, wasting, and fasciculation of the tongue. Pseudobulbar palsy (upper motor neuron) manifests with brisk jaw, jerk, dysarthria, and emotional lability. Cervical-onset is associated with upper-limb symptoms, either bilateral or unilateral, affecting upper and/or lower motor neurons. Proximal weakness presents problems affecting patient’s daily activities such as hair washing, combing, etc., and difficulties with precise movements such as catching and manipulating small objects. Lumbar-onset is linked to lower motor neurons and symptoms include fasciculations, wasting, and weakness [10] (Table 1). 

## 2. Risk Factors

**Figure 1:**
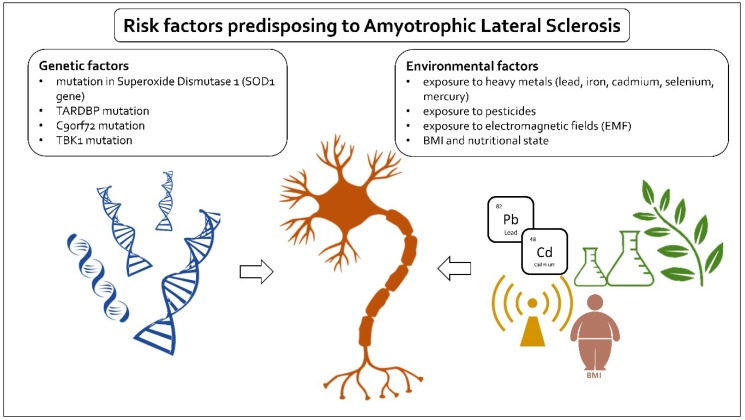
Schematic depiction of risk factors in ALS. ALS risk factors can be divided into two main categories: genetic and environmental. Well-documented genetic factors include mutations in superoxide dismutase 1 (*SOD1* gene), mutation in TAR DNA binding protein (TARDBP), a hexanucleotide repeat expansion (GGGGCC) in the first intron of the *C9orf72* gene, and mutations in TANK-binding kinase 1 (TBK1). The researched environmental risk factors are BMI and nutritional state and exposure to heavy metals, pesticides, and electromagnetic fields.

Nowadays, there is a lot of research on the probable risk factors for ALS. Older age, male sex, and a family history of ALS have all been established as risk factors [11] (Figure 1). 

Genetic mutations leading to the onset of fALS account for only 10–15% of all ALS cases [12], and the remaining 85–90% of all cases are of unknown etiology [13]. Though the exact pathogenesis remains unclear, it is generally accepted that both genetic and non-genetic contributors play a role in ALS pathogenesis. New technologies like gene mapping have allowed us to detect about 30 gene mutations involved in ALS pathogenesis. The first mutation implicated in ALS, discovered in 1993, was a mutation of the superoxide dismutase 1 (*SOD1*) gene [14]. Nowadays, more than 20 different genes have been implicated in fALS, sALS, or both [15]. Mutations in genes like *SOD1*, *TARDBP* [16], *FUS* [17], *OPTN* [18], *VCP* [19], *UBQLN2* [20], *C9orf72* [21], and *TBK1* contribute to the development of this disorder [22]. Aside from genetic causes, a number of environmental factors likely contribute to the development of ALS. In this review, we would like to provide the most common and important genetic and environmental factors with predispositions to ALS. 

## 3. Genetic Factors

### 3.1. Mutations in Superoxide Dismutase 1 (SOD1 Gene)

*SOD1* mutations have been the first mutation reported to contribute to the development of ALS and account for 15–20% of all fALS [23] and 3% of sALS [24] cases. 

The *SOD1* gene is located on chromosome 21q22.11 and encodes the monomeric SOD1 protein, which consists of 153 amino acids and has a mass of 16 kDa. [25]. SOD1 eliminates free radicals, transforming free superoxide radicals into molecular oxygen O_2_ and H_2_O_2_ [26]. At present, more than 180 mutations have been found and characterized, but not all of them are pathogenic [27]. SOD1 is a cytosolic enzyme, but it also is present in lysosomes, the nucleus, and mitochondria [28,29]. D90A, A4V, and G93A are the most common mutations found in the *SOD1* gene. A Glycine 93 change to alanine (G93A) is the rarest yet most researched of these three mutations. G93A may be inherited as a dominant or recessive trait, but in most of cases, it is recessive [30]. G93A was the first mutation studied in a transgenic mouse model shown to cause motor neuron syndrome. On the contrary, the most common SOD1 mutation found in North America population of ALS patients is an alanine-valine change at codon 4 [31]. SOD1 mutation results in the accumulation of mutant SOD1 aggregates in mitochondria, disrupting their function, and triggering the accumulation of reactive oxygen species (ROS), subsequently leading to mitochondrial damage and neuronal death [32,33,34]. 

### 3.2. TARDBP Mutation

TAR DNA binding protein 43 kDa is a transactivate response nuclear protein. It is a major component of neuronal cytoplasmic inclusions (NCIs) in sALS [35]. Mutations in TAR DNA binding protein (TARDBP) have been identified in a small number of patients, accounting for 4% of fALS and 1% of sALS cases [36]. TAR DNA-binding protein 43 (TDP-43) as well as FUSED IN SARCOMA (FUS) are RNA-binding proteins that have been shown to mislocalize from the nuclear to the cytoplasmic compartment when mutated. One of the plausible mechanisms of their action is a loss of normal processing of their target RNA [37,38], likely exerted by the prion-like domains present in both proteins. TDP-43 is thought to be a transcriptional regulator and displays a significant specificity for binding with the common microsatellite region (GU/GT) in DNA and RNA [39]. In ALS, a loss of functional TDP-43 has been observed. This loss has detrimental effects on RNA metabolism and has been linked to the cytosolic accumulation of protein aggregates that leads to neurotoxicity [40]. 

### 3.3. C9orf72 Mutation

A hexanucleotide repeat expansion (GGGGCC) in the first intron of the *C9orf72* gene has been linked to the pathogenic mechanism of two diseases: familial amyotrophic lateral sclerosis (fALS) and frontotemporal dementia (FTD). Mutations of *C9orf72* is present in about 5% of all sALS and 30% of all fALS cases, making it the most important mutation associated with ALS pathogenesis. In FTD, it has been detected in 25% of all patients with familial ALS, underlining the importance of the hypothesis that classical ALS and FTD represent the extremes of a single mutation spectrum [41]. In control populations, the number of repeats is 2–30 while in ALS patients it might be more than hundred or thousand repeats [42] per person. The inheritance of this mutation is autosomal dominant [43]. A common genetic cause of fALS and FTD was proposed in 1991 by linkage analyses. Similarly, genome-wide association studies (GWAS) also pointed to a common underlying genetic factor located in chromosomal region 9p21.2 [44]. For chromosome 9 open reading frame 72 (*C9orf72*), bulbar onset has been observed more frequently than others. Unfortunately, it is not clear whether the same is true for *C9orf72*-associated diseases [45]. In ALS patients with *C9orf72* and *TDP43* mutations, executive dysfunction, dementia, and cognitive impairment have been observed in more than 50% of all cases [46,47]. Patients presenting a hexanucleotide repeat expansion are distinguished by a lower age at onset and shorter survival time [48].

### 3.4. TBK1 Mutation

TANK-binding kinase 1 (TBK1) is a recently identified gene with four domains: an α-helical scaffold dimerization domain (SDD), a kinase domain (KD), a ubiquitin-like domain (ULD), and a C-terminal domain. The KD is responsible for kinetic activity and also interacts with the SDD. The SDD interacts with the ULD and C-terminal ends. The dimerization of TBK1 is possible because of three domains interacting and enables binding to proteins like optineurin, which plays a role in autophagy [49,50].

The TBK1 protein is a multifunctional kinase, which is known to bind to and phosphorylate a series of proteins involved in innate immunity and autophagy [51], including optineurin (OPTN) and p62, both of which have been implicated in the process of ALS [52,53]. TBK1 participates in cell growth [54] and proliferation as well as in bacterial clearance [55,56]. Mutations in TBK1 might occur as a loss-of-function or missense mutations, causing loss of interaction with its adaptors or reduction of protein expression, respectively [57,58]. The impaired autophagy in ALS might be caused by generating premature stop codons that lead to haploinsufficiency and nonsense-mediated mRNA decay. Unfortunately, the mechanism of the missense mutation and pathogenicity remains unexplained [59]. Cui and collaborators conducted a meta-analysis which showed that the TBK1 LoF mutations are less frequent than missense mutations in ALS/FTD patients. They also noticed that both of these mutations are less common in Asian than in European patients. They suspected that the pathology of ALS was not only a consequence of mutation of TBK1 itself but also other mechanisms of pathogenesis that has not yet been identified [60].

## 4. Environmental Factors

In recent years, progress has been made in deciphering some of the environmental factors linked to the development of the sporadic form of ALS. Growing evidence suggests that a number of environmental factors such as an exposure to heavy metals, pesticides, head trauma, electromagnetic fields, high BMI and nutritional state, β-N-methylamino-l-alanine (BMAA), and even physical activity (affecting professional football players in particular) contribute to the onset of the disease [61]. 

### 4.1. Lead and Other Heavy Metals

The exposure to heavy metals, like lead, iron, cadmium, selenium and mercury, has been studied for many years. According to previous studies, lead may be one of the major environmental factors leading to the development of ALS, as indicated by lead exposure measurements in patients with ALS. Concerns about the association between lead exposure and ALS started about 50 years ago with reports of ALS patients who have developed the disease after a prior lead-linked exposure. Wang and colleagues showed nine case-control studies in which antecedent exposure to lead in occupational environments led to a high risk of ALS. However, they pointed out that in recent years, lead pollution has been reduced and, because of that, lead should not be considered causal of many ALS cases at present [5,62]. 

Unfortunately, the results are inconsistent. Trojsi and colleagues have observed that the potential role of several heavy metals as contributors to molecular mechanisms leading to motor neuron degeneration has been widely explored but only partially characterized. Unfortunately, results of the relationship between lead exposure and genetic susceptibility were contradictory and unconvincing [63]. In several ALS patients, polymorphisms on metallothionein (MT), transcription factor (MTF-1), and glutathione synthetase (GSS) genes were detected [64]. Polymorphisms have also been found in other genes such as for delta-aminolevulinic acid dehydratase (ALAD) or vitamin D receptor (VDR), which are likely involved in determining genetic susceptibility. However, the evidence of correlation between the observed polymorphisms and the development of the disease has been contradictory [65,66]. 

### 4.2. BMI and Nutritional State

BMI is a measure defined as weight in kilograms divided by height squared in meters. It shows whether people have proper weight for their height. In ALS, weight loss is normal due to muscle mass loss and a reduction in body fat mass [67]. Paganoni and colleagues found that patients with a BMI between 30–35 had better survival outcome than those with a BMI above 35 or below 30 [68]. Recent studies show that higher pre-morbid BMI not only predicts a better result in ALSFRS-R (ALS Functional Rating Scale) [69] but, as found by O’Reilly and colleagues, increased BMI in earlier life is also associated with a lower incidence of ALS [70]. It is known that reduced body mass contributes negatively to ALS [71,72]. Poorer ALSFRS-R is associated with reduced deposition of subcutaneous fat mass [73]. Thus, caloric restriction can cause faster onset of clinical symptoms of the disease and a shortened life [74]. 

### 4.3. Pesticides

The known class of pesticides are insecticides, fungicides, herbicides, and rodenticides. The paths of penetration include, but are not limited to, oral, dermal, and inhalation routes. Pesticides induce oxidative stress, mitochondrial dysfunction, α-synuclein storage, and neuronal loss, and at higher doses, they are likely contributing to the development of ALS. However, pesticides have been implicated in the pathogenesis of other neurodegenerative diseases as well [75], thus making it harder to pinpoint their involvement in ALS. It should be also noted that genetic predisposition combined with long-term consequences of exposure to pesticides also play a role in the development of the disease. Bozzoni et al. mentioned the importance of genetic predisposition and induced damage by the long-term effects of pesticide exposure. In their studies, they underlined the role of the paraoxonase gene cluster (*PON1*). *PON1*, the most intensively studied gene, encodes A-esterase paraoxonase-1, an enzyme that is able to hydrolyze organophosphate pesticides. This ability depends on some genetic variants. Reduced detoxifying activity is determined by *PON1* genetic polymorphisms. Therefore, *PON1* mutations may predispose ALS by reducing pesticide hydrolysis and promoting oxidative stress processes [76]. In Slowik’s study, an association between ALS risk and the *PON1* (Q192R) and *PON2* (C311S) gene has been reported [77]. Ticozzi et. al. found eight heterozygous rare variants in nine fALS and three sALS cases, and five of these variants were not found in any of the control samples, suggesting a link between these variants and the development of ALS [78]. They proved a close dependence between *PON1* and ALS. This association should not be ignored, because not all *PON1* variants have been found.

It has been also found that in the central nervous system, *PON2* genes are down-regulated [79], indicating that pesticides pose a real risk and likely play a role in the development of ALS and other neurodegenerative disorders such as Parkinson’s and Alzheimer’s disease [80]. 

### 4.4. Electromagnetic Fields (EMF)

Some studies have shown that occupational or residential exposure to electromagnetic fields (EMFs), especially extremely low-frequency electromagnetic fields (ELF-EMFs), and electric shocks might be a causal factor of ALS. ELF-EMFs have frequencies ranging from 3 Hz to 3000 Hz. Some professions, like electricians, electrical and electronic equipment repairers, power plant operators, telephone installers and repairers, or train drivers, are constantly exposed to EMFs and ELF-EMFs [81,82]. In the 1990s, researchers found a relationship between the increasing risk of motor neuron disease and occupations related to electricity [83,84]. From then on, many studies on the effects of occupational ELF-EMF exposure and the incidence of ALS have been published [85]. Previous observations showed positive associations between occupational ELF-EMF exposure and ALS risk [86], but a few studies did not confirm this association [87,88]. Zhou and collaborators found conducted a meta-analysis and found a slight but significant ALS risk increase among those with job titles related to relatively high levels of ELF-EMF exposure [89]. 

### 4.5. Oxidative Stress as a Common Aspect in Number of Environmental Risk Factors

It has been suggested that some of environmental risk factors in ALS have a common denominator that is oxidative stress, a condition facilitated by any factor that favors a pro-oxidative state [90,91]. Oxidative stress is a long-studied aspect of biochemical processes in aerobic organisms that use oxygen in converting energy coming from nutrients into adenosine triphosphate. It has been named a potential “Mother” of many human diseases, contributing to a large number of human pathologies like diabetes, cardiovascular, inflammatory diseases, amyloidosis, autoimmune processes, and cancer [92]. The state of oxidative stress arises when the capability of the cell to maintain balance and eliminate excessive reactive oxygen species (ROS) is compromised by an excess number of ROS or hindered mechanisms of their removal, which results in a homeostasis break. This leads to the damage of cell organelles and components, such as lipids, proteins, and DNA, which if unrepaired, can result in cell death [93]. 

The mechanism of cellular oxidative stress depends on particular risk factors. Heavy metals such as lead, cadmium, and mercury are redox-inactive, which means they deplete a cell’s antioxidants, principally thiol-containing antioxidants, and enzymes. [94]. Organophosphorus pesticides affect oxidative stress by influencing total thiol molecules and alteration in total antioxidant capacity, and additionally also by cell membrane lipid peroxidation [95]. Studies with electromagnetic fields, another environmental risk factor, showed that exposure to extremely low frequency electromagnetic waves in vitro resulted in a larger quantity of cellular reactive oxygen generated [96]. In vivo research demonstrated that similar exposures not only induced oxidative stress but also disabled antioxidant properties within a cell [97].

## 5. ALS Treatments

### 5.1. Existing Disease-Modifying Treatments

There is no known cure for ALS, but there are at present two recognized treatments that are being classified as disease-modifying. The longest available is riluzole [98]. Since its approval in 1995 until recently, it was the only ALS targeting treatment in existence. Riluzole slows the progression of ALS, but its efficacy is modest, with survival benefit of approximately 3 months and 9% gain in the probability of surviving one year [99]. Riluzole is an anti-glutamatergic agent, targeting excitotoxicity that is thought to play a role in ALS pathophysiology. The knowledge of the exact mechanism of riluzole action in ALS treatment is not well established. There are few known pathways by which it can contribute to a decrease in excitotoxicity: presynaptic inhibition of glutamate release, slowing inactivation of potassium channels, inactivation of voltage-gated sodium channels, inhibiting protein kinase C, and interfering with intracellular events following transmitter binding at excitatory amino acid receptors [100,101]. There are inconsistent reports regarding the effects of riluzole across different phases of the disease. Earlier findings [102,103,104,105] suggested that the most beneficial effects for patients are in the early stages of ALS; however, a recent study by Fang and colleagues [106] showed that riluzole prolongs survival in the last clinical stage of ALS (survival time in stage 4 was longer for patients receiving 100 mg/day riluzole than for those receiving placebo; hazard ratio [HR] 0.55, 95% CI, 0.36–0.83; log-rank p = 0.037). That means riluzole’s efficiency demonstrates itself with an already mostly depleted population of dying and dead motor neurons. Some authors [99], when comparing results of earlier findings with Fang’s data, suggest that riluzole might affect or activate different therapeutic pathways depending on disease stage, with modulation of excitotoxicity as an early transient effect, followed by molecular pathways becoming more involved later. 

The second disease-modifying treatment that has been only recently accepted for ALS patients in USA [107], and is awaiting approval by the European Medicines Agency [108], is edaravone. It is a strong antioxidant agent, being reported to eliminate lipid peroxides and hydroxyl radicals. The mechanism of edaravone action in ALS is uncertain as it is in a case of riluzole. Presumably, the drug mitigates oxidative injury in neurons and neighboring glia that are at risk for degeneration in ALS [100]. The drug proved to be beneficial, exposing effective inhibition of the motor function deterioration in early stage ALS patients, as shown in a multicenter phase III study focused on that specific subgroup conducted in Japan with 137 subjects [109]. Although in the USA, edaravone has been approved for treatment of all ALS patients, its FDA application in 2017 focused on the potential benefit in early disease stages, i.e., within 2 years of onset, in patients with a forced vital capacity of >80%. It is estimated that this population of individuals with ALS typically accounts only for 7% of all cases [110]. Recent reports, like provided by Abraham and colleagues [111] from research conducted in a real-life setting at Tel Aviv Sourasky Medical Center between May 2017 and January 2018 and on data retrospectively collected for 22 patients who opted for treatment with edaravone (compared to 71 untreated ALS patients), seem to confirm that edaravone is not effective in unselected ALS patients.

### 5.2. Researched Treatments

**Figure 2:**
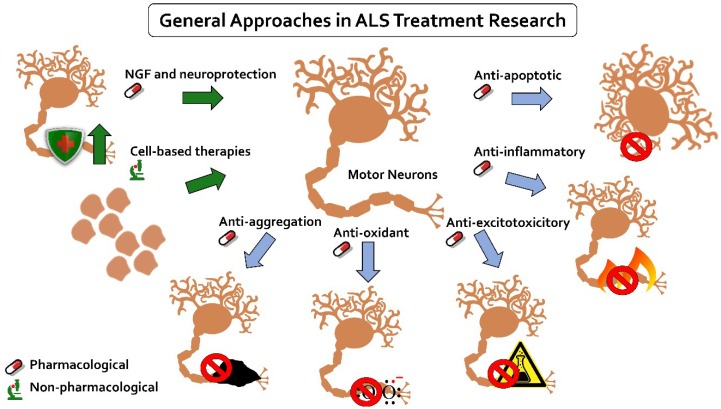
Overview of main approaches to ALS treatment. The therapies studied can be divided into seven general groups, mainly based on pathophysiological models of the disease. The groups include anti-apoptotic, anti-inflammatory, anti-excitotoxicitory, anti-oxidant, anti-aggregation, neurotrophic growth factor, and neuroprotective approaches, which all can be classified as pharmacological interventions. Additionally, a separate non-pharmacological group of stem cell therapies is discussed.

A number of therapies have been or are being studied on different stages of preclinical and clinical assessments. They can be categorized into groups based on pathophysiological models of the disease [101] (Figure 2).

#### 5.2.1. Anti-Apoptotic

This group of therapies focuses on processes such as mitochondrial impairments of injured motor neurons and abnormal calcium handling leading to apoptotic cascade. Trials in this domain include large studies such as the one on Dexpramipexole [112,113], with failed outcomes [114,115], and also more recently, preliminary reporting on Ursodeoxycholic acid and Tauroursodeoxycholic acid use [116,117], both with moderately positive outlooks.

#### 5.2.2. Anti-Inflammatory

It has been shown that neuroinflammation processes associated with reactive astrocytes, microglia [118,119], and infiltrating T-lymphocytes and microphages [120] play an important role in ALS neurodegeneration. Studies of possible treatments in this area include a phase III study of recombinant human erythropoietin (rhEPO) [121] conducted on 208 patients in Italy in which the progression course of ALS was not been changed, as well as a randomized phase II trial of the NP001 immune regulator, which demonstrated a slowing of progression of the disease in the high-dose group of patients with greater inflammation (however, the latter study lacked precision to be conclusive about the overall effectiveness of NP001) [122]. The drug which showed the greatest potential in this category is Masitinib, which demonstrated a slowing of ALS progress by 27% in preliminary results in a recent phase III study when it was combined with riluzole treatment [123]. 

#### 5.2.3. Anti-Excitotoxicitory

Glutamate is the main modulator of excitotoxicity. In ALS, excitotoxicity is derived from excessive glutamate release combined with changes in postsynaptic glutamate receptors and glutamate transport. Loss of EAAT2 transporter receptors in ALS is considered a primary factor in leaving patients with decreased glutamate transport capacity, which leads to increased glutamate levels in their CSF [124]. The main treatment targeting the excitotoxicity pathophysiological pathway is the earlier mentioned riluzole. New compounds, with the latest having reached phase III trial (ceftriaxone), have been probed in this domain with negative outcomes.

#### 5.2.4. Anti-Oxidant

Oxidative stress is suggested as one of the prominent factors possibly playing a crucial role in the pathogenesis of ALS. It is highlighted as a potential common denominator of multiple ALS risk factors [125]. This pathophysiological pathway is targeted by the second-available ALS approved treatment edaravone [126]. Besides this, the antioxidant considered to be the most promising for treating ALS is AEOL 10150, a small molecule that catalytically consumes reactive oxygen species and reactive nitrogen species. This antioxidant was put under evaluation in a clinical trial with results reported in 2009 [127]. This open-label, small trial showed AEOL 10150 to be well tolerated and safe. However, there has been a lack of subsequent efficacy measurements and oxidative biomarkers in ALS patients reported till now [125]. 

#### 5.2.5. Anti-Aggregation

The phenomenon of cellular protein aggregation is a known feature of ALS [128]. Mutations in SOD1 leads to conformational instability, disorder, and the formation of SOD1 protein aggregates. It is thought that preventing those cellular aggregates may increase motor neuron survival [129]. Another form of pathologic aggregates that characterizes affected neurons in patients with amyotrophic lateral sclerosis is represented by TDP-43, a primarily nuclear RNA-binding protein. 

A recently discussed compound that shows inhibition of toxic misfolded SOD1 amyloid aggregates is the macrophage migration inhibitory factor (MIF). MIF alters the typical SOD1 amyloid aggregation pathway in vitro and, instead, promotes the formation of disordered aggregates [130]. The TDP-43 aggregation antagonizing agent is an acridine derivative, 4,5-bis{(N-carboxy methyl imidazolium)methyl}acridine] dibromide. Findings indicate that it separates the adjoining low-complexity domains of consecutive TDP-43 monomers, thus disrupting the formation of pathologic aggregates [131]. Both agents are proposed as potential therapeutic candidates in the treatment of ALS.

#### 5.2.6. Neurotrophic Growth Factors and Neuroprotection

The main aspect of pathophysiology of ALS is the degeneration of neuronal tissue. Instead of cutting the pathological process paths, neurotrophic and neuroprotection treatment research focuses on drugs that could stimulate the repair of damaged neurons and promote the growth of new ones. Substances such as 7,8-dihydroxyflavone, which increased the survival rate of affected neurons in mouse models of ALS [132], or glycoprotein non-metastatic protein B (GPNMB) [133], which showed protective effects against mutant TDP-43 stress [134], demonstrate good potential for neuroprotective treatments in ALS.

### 5.3. Cell-Based Therapies

There are many studies on cell-based therapies for the treatment of neurodegenerative disease. Stem cell applications have provided researchers with a powerful tool for discovering of new mechanisms and therapeutic agents [135]. ALS is a slow but steadily progressing disease. Cell-based therapies aimed at delaying progress and symptoms of disease have been proposed as an ultimate source for the regeneration of MNs [136].

#### 5.3.1. Glial-Restricted Precursors

Astrocytes maintain a major role in the structural health of neurons. Astrocytes control the metabolism and homeostasis of synaptic activity and neural cells and are responsible for the brain environment and the micro-architecture of nervous system. Astrocytes are involved in all chronic neurodegenerative disorders. It is known that transplantation of astrocytes and glial-restricted precursor (GRP)-derived astrocytes (hGDAs), which are lineage-restricted precursors capable of differentiating into astrocytes after transplantation, promoted neural regeneration processes [137]. Studies showed that GRPs largely differentiate into astrocytes after transplantation and their therapeutic benefit is probably due to astrocyte-derived functions [138,139]. Haidet-Phillips and colleagues showed that in an environment characterized by active motor neuron degeneration, the hGRPs did not show any signs of astrocytosis or morphological transformation after transplantation. On the other hand, they demonstrated an upregulation of EAAT2, which is responsible for the transport of the majority of the extrasynaptic glutamate in the central nervous system. It can be concluded that increased levels of EAAT2 indicate a therapeutic potential for hGRPs [140].

#### 5.3.2. Neural Progenitor Stem Cells

Neural stem cells (NSCs) are progenitor cells located in both the immature and mature central nervous system (CNS) [141]. NSCs secrete extracellular vesicles (EVs) that communicate with the host’s immune system [142]. Components like lipids, proteins, and nucleic acids mediate a neuroprotective and immunomodulatory effect and, when they go through the blood brain barrier (BBB) and get into nerve cells during the inflammatory process, they could heal these cells. Unfortunately, cellular and molecular mechanisms have not yet been fully explained. NSCs are self-renewing and multipotent and are characterized by the ability to give rise to neuronal cells. Because of these features, NSCs are becoming a promising therapy for ALS patients. Animal models revealed that motor neuron (MN) degeneration activates endogenous NSCs in the CNS to multiplicate, migrate, and stimulate neurogenesis in the spinal cord as a natural defense mechanism. Nevertheless, the amount of these cells does not seem to be enough to fight with the advancing degeneration connected with ALS [143]. Mazzini and collaborators found that NSCs support earlier-damaged motor neurons through their neurotrophic (humoral) and anti-inflammatory activities. Stem cells produce and secrete immunomodulatory molecules, which regulate cell migration, growth, and differentiation and lead to the process of neurogenesis and angiogenesis [144]. There is no doubt that using the exceptional characteristics of NSCs in ALS therapy can bring positive results. 

### 5.4. Gene Therapy Strategy

The progress of medicine brings new and alternative opportunities for the treatment of so-far-incurable neurodegenerative diseases such as ALS. 

Attractive therapeutic strategies might be with using antisense oligonucleotides (ASOs) and RNA interference approaches in neurological diseases [145]. It has been shown that ASO therapy in rodent models of ALS with SOD1 mutations significantly delays disease progression [146]. Another useful approach is through the delivery of GDNF (glial cell line derived neurotrophic factor) by viral vector AAV serotype 9 (AAVs9). AAVs9 has a high gene transduction and transfer to astrocytes and motor neurons [147]. The strategy of using AAV9-SOD1-shRNA reduced disease onset and extended survival in SOD1 mice and also in SOD1 rats [148]. Thomsen and colleagues treated SOD1 rats by systemic injection in the tail vein. They proved that this injection delayed forelimb paralysis onset and brought some functional improvement. However, it should be mentioned that these rats showed slower weight gain, reduced activity, and had problems with working memory [149]. The problem is that SOD1 mutations account for about 2–5% of ALS cases and, therefore, more therapeutic strategies are needed. 

More frequently occurring than the SOD1 mutation is *C9orf72*. Martier et al. in their study tried to reduce the gain of toxicity caused by hexanucleotide repeat expansion. They used AAV (adeno-associated virus) 5-miC to reduce repeat-containing transcripts that cause a gain of toxicity by accumulation of RNA foci in the nucleus and cytoplasm of affected cells. The post-mortem analysis of gene-therapy-treated brains of treated ALS mice showed a reduction in nuclear RNA foci. The results of the study indicate that the therapy used may mitigate the RNA-mediated toxicity in the cell nucleus in ALS patients [150]. In conclusion, scientists are continually looking for effective therapies for ALS treatment and it seems that, among others, gene therapies show a lot of promise and brings many positive aspects in tackling ALS progression and treatment.

## 6. Factors in “ALS Reversal” Cases

In addition to the risk factors in ALS, looking at aspects accompanying possible “ALS reversal” cases might prove beneficial. Harrison and colleagues, with cooperation of The Pooled Resource Open-Access ALS Clinical Trials Consortium, conducted a study aimed at identifying factors that differed “ALS reversal” cases, which is defined as patients with the disease that stopped progressing and regained significant motor functions, from patients with typical ALS progression [151]. Of the initial 89 possible reversals identified, 36 cases were included in the analysis. Cases and controls were compared using descriptive statistics. It was revealed that reversal cases were more likely to be male, have limb onset of disease, and have a faster initial progression. Co-morbidities were investigated, which showed that the reversal population had a higher prevalence of myasthenia gravis (MG) and purely lower motor neuron disease. Treatments used by patients at the time of their maximum improvements were taken into account. The analysis demonstrated, with age and site of onset controlled, that the odds of taking substances such as curcumin, azathioprine, copper, glutathione, vitamin D, and fish oil was significantly greater for these cases than in controls. Further studies aiming at scrutinizing the genome of reversal cases are planned.

## 7. Conclusions

ALS is considered a multifactorial disease with environmental and genetic risk factors. A lot of genes are involved in the possible pathophysiological pathways. More and more new genes likely contributing to ALS pathogenesis have been discovered in recent years. The plausible hypothesis states that ALS is the ultimate result of either a single gene mutation or of polygenic pathogenetic pathways with yet to be determined influence from environmental factors. Despite 160 years of recorded research history in ALS and more than 200 clinical trials [90], there has been no cure developed for ALS, and only two modestly effective disease-modifying treatments that slow its progression are available. There are a number of distinctive therapy research approaches being utilized, including emerging investigations into stem cell treatments. Treatments of combined origin might soon be developed to address the complex and multifaceted origin of this incapacitating condition.

## Figures and Tables

**Table 1:** Clinical phenotypes, symptoms, and prognosis of amyotrophic lateral sclerosis (ALS).

Clinical Phenotypes	Regions	Symptoms	The Prognosis
**Limb-onset**	UMN	Spasticity, weakness, and brisk deep tendon reflexes;	Overall survival is 5–8 years
	LMN	fasciculations, wasting, and patients present with gradually ascending distal weakness.	
**Bulbar-onset**	UMN	Spastic dysarthria, which is characterized by slow, labored, and distorted speech, often with a nasal quality;	2 to 4 years, and depends on the timing of respiratory and limb involvement.
	LMN	tongue wasting, weakness, and fasciculations, accompanied by flaccid dysarthria*, and later dysphagia.	
**Primary lateral sclerosis**	Pure UMN	It is characterized by an ascending spastic tetraparesis with involvement of speech in the majority by 3 years, urinary urgency;	A slowly progressive condition, with survival for decades.
**Progressive muscular atrophy**	Pure LMN	The least well-defined subtype of ALS. Asymmetrical weakness and wasting, often in the legs, which coalesces into four limb lower motor neuron involvement.	About 5 years
**ALS-frontal lobe dementia syndrome** **(frontotemporal lobar degeneration, FTLD)**	UMN, LMN, and brain cortex	Presentation with frontotemporal dementia. Later developing signs of MND.	An overall survival of less than 3 years.

UMN-upper motor neurons, LMN - Lower motor neuron.
